# Effects of rosemary essential oil on the milk production and fatty acid profiles of ewes fed oat hay or silage

**DOI:** 10.5194/aab-67-177-2024

**Published:** 2024-04-17

**Authors:** Samir Smeti, Hadhami Hajji, Margalida Joy, Naziha Atti

**Affiliations:** 1 University of Carthage, INRA-Tunisie, Laboratoire de Productions Animales et Fourragères, Rue Hédi Karray, 2049 Ariana, Tunisia; 2 University of Gabes, IRA Médenine, Laboratoire d'élevage et de la faune sauvage, Route du Jorf, 4119 Medenine, Tunisia; 3 Centro de Investigación y Tecnología Agroalimentaria de Aragón (CITA), Instituto Agroalimentario de Aragón – IA2, CITA-Universidad de Zaragoza, Avda. Montañana 930, 50059 Saragossa, Spain

## Abstract

Milk and dairy products are among daily-consumed foods in most countries. However, milk production and characteristics depend mainly on animal feeding and additives. The basic feeding corresponds to green or conserved forage and concentrate. The aim of this study was to investigate the effects of a conserved oat forage form (hay, H, or silage, S) and rosemary essential oils (REO) as additives on milk production and the fatty acid (FA) profile of Sicilo-Sarde dairy sheep. Forty-eight lactating Sicilo-Sarde ewes were ranked into four homogenous groups. Two groups were fed oat hay (H) and the other group oat silage (S) ad libitum. All the ewes were supplemented with 600 g d ${}^{-1}$ of concentrate. Within each form of forage, one group of ewes received a control concentrate, and the other group received the REO concentrate.

The milk yield was not affected by REO intake but tended to be higher ( P=0.07 ) for oat hay than for silage (570 vs. 510 mL d ${}^{-1}$ ). The milk protein content was greater for oat silage than hay diets (5.8 vs. 5.3 %), given that the silage form contains more crude protein. In addition, the silage form of forage improved the milk FA profile, with higher C18: 3n-3 (0.70 vs. 0.45 %) long-chain FA and polyunsaturated FA (PUFA) n3 proportions and a lower PUFA n-6/n-3 dietetic ratio ( P<0.05 ). The milk of ewes receiving REO contained a lower percentage of vaccenic acid and n-6 PUFA but a higher percentage of n-3 PUFA and consequently lower n6/n3 (2.56) and PUFA / SFA ( P<0.05 ) ratios. In conclusion, REO could be recommended for dairy ewes fed silage to improve the nutritional quality of their milk for human consumption.

## 1 Introduction

Milk stands as one of the most valuable agricultural commodities on a global scale. Dairy animals offer significant advantages to rural communities, serving as a consistent source of both sustenance and income for farmers (Wanapat et al., 2015). Notably, milk, in line with its widespread production, enjoys consumption in nearly every corner of the world, as reported (FAO, 2017). The perception of milk and dairy products has evolved beyond simply being provisions for addressing hunger and malnutrition. They now play a pivotal role in promoting human health and overall well-being, aligning with the growing trend of health-conscious consumers actively seeking nutritious dietary options (Guthrie et al., 2015). Milk is renowned for its high nutritional value, attributed to its rich content of proteins, fats, essential minerals such as calcium, magnesium, and selenium, as well as vitamins B ${}_{5}$ and B ${}_{12}$ (Kailasapathy, 2015; Cabiddu et al., 2022). Nevertheless, it is important to note that milk production and overall animal product quality are subject to various influencing factors, with a particular emphasis on animal nutrition, which relies on a combination of green forage, conserved feed, and concentrated supplements (Smeti et al., 2014; Yagoubi et al., 2018; Cabiddu et al., 2021). Among natural additives, certain plant compounds, particularly essential oils (EO), have garnered attention for their potential benefits (Dorantes-Iturbide et al., 2022). These EO are known to have positive effects on rumen microbes and can influence various microbial processes in the rumen (Cardozo et al., 2005). Additionally, the efficacy of some EO in combating pathogens has been verified (Sharifi-Rad et al., 2017). In this context, incorporating certain additives into diets has the potential to enhance economic productivity by improving both the dairy production and milk quality of ewes (do Nascimento et al., 2022). It is worth noting that EO are frequently employed as additives to enhance meat production and quality (Chaves et al., 2008; Smeti et al., 2021; Dorantes-Iturbide et al., 2022). However, their utilization in the context of dairy sheep remains relatively limited (Kalaitsidis et al., 2021). Nevertheless, there is a growing consumer demand for dairy products that offer potential health benefits. These products are often associated with the category of polyunsaturated fatty acids (PUFAs), including conjugated linoleic acid (CLA) and particularly n-3 PUFA.

The consumption of dairy products rich in n-3 PUFA, such as eicosapentaenoic acid (EPA) and docosahexaenoic acid (DHA), holds the potential to reduce the risk of cardiovascular diseases, modulate autoimmunity, and mitigate inflammatory disorders (Calder, 2015; Roopashree et al., 2021). As a result, there is a growing interest in exploring natural alternatives to increase the production of PUFA in the forestomach, consequently elevating its levels in milk, as emphasized by Nudda et al. (2020). On the other hand, the positive impact of green forage, whether through grazing or stall feeding, on the quality of both meat and milk, with particular regard to PUFA content, has been well-documented (Agradi et al., 2020; Nudda et al., 2020).

Moreover, while the FA profile of Sicilo-Sarde sheep milk has been extensively explored under conditions of green forage grazing (Atti et al., 2006; Maamouri et al., 2019), it is imperative to acknowledge the significant influence of seasonal variations on the availability of green forage in various regions worldwide. This seasonal dependence often leads to a shortage of green forage for a considerable portion of the year, necessitating the feeding of animals with conserved forage in the form of hay or silage (Sitzia et al., 2015; Agradi et al., 2020). Therefore, based on this contextual background, the primary objective of the present study is to assess whether dietary feeding with both forms of conserved forage (hay and silage) and the addition of rosemary EO (REO) can indeed impact dairy sheep production and the composition of fatty acids in their milk.

## 2 Material and methods

The experiment was carried out at the experimental farm Lafareg (Beja) of the National Institute of Agricultural Research of Tunisia (INRAT) situated in the subhumid region of Tunisia, characterized by an altitude of 222 m and geographic coordinates of 36.43° N and 09.12° E. The area typically experiences an average annual rainfall of 650 mm. Throughout the duration of the experiment, the mean temperature and humidity levels were approximately 17.5 °C and 65 %, respectively.

### 2.1 Experimental design

Forty-eight lactating Sicilo-Sarde ewes were individually housed in pens measuring 1.5 m × 2.5 m and had unrestricted access to water. They were ranked into four homogenous groups based on age (4.2 ± 1.2 years), body weight (43.1 ± 4.7 kg), and milk production of the first week (538 ± 102 mL d ${}^{-1}$ ). Two of these groups were provided with oat hay (H), while the remaining groups received oat silage (S) offered ad libitum. All the ewes received a supplement of 600 g d ${}^{-1}$ of concentrate. The concentrate and forage were separately presented to the ewes and their single lambs in two feed bins in the morning at 07:00 and in the afternoon at 16:00. Within each type of forage, one group of ewes was provided with the control concentrate (C), which consisted of a mixture of barley (800 g kg ${}^{-1}$ ), soybean meal (180 g kg ${}^{-1}$ ), and a mineral vitamin supplement (20 g kg ${}^{-1}$ ). The experimental concentrate (REO) contained the same mixture as the control, along with an additional 0.6 g kg ${}^{-1}$ of rosemary essential oil. The chemical composition and fatty acid profile of the experimental feeds are detailed in Table 1. Information regarding the composition and dosage of the REO is detailed in a previous publication (Smeti et al., 2015).

**Table 1:** Chemical composition and fatty acid (FA) profile of the experimental feed.

	Forage		Concentrate ${}^{*}$	
	Hay	Silage	Control	REO
Dry matter (g kg ${}^{-1}$ )	900	320	902	904
Organic matter (g kg ${}^{-1}$ DM)	901	931	799	811
NDF (g kg ${}^{-1}$ DM)	601	621	225	263
Crude protein (g kg ${}^{-1}$ DM)	45	71	152	164
Fatty acid composition (% of the total FAME)				
Myristic acid (C14:0)	3.5	5.8	–	–
Palmitic acid (C16:0)	28.6	32.6	12.3	12.8
Palmitoleic acid (C16:1)	0.1	0.2	0.2	2.1
Margaroleic acid (C17:1)	0.1	10.9	0.1	2.0
Stearic acid (C18:0)	5.7	8.0	0.2	0.2
Oleic acid (C18:1)	14.2	15.0	23.5	21.1
Linoleic acid (C18:2 n-6 c9,c12)	21.9	8.3	48.3	50.1
α -Linolenic acid (C18:3 n-3 )	15.1	8.9	2.6	3.6
γ -Linolenic acid (C18:3 n-6 )	0.2	4.5	1.8	0.6
Saturated fatty acid (SFA)	41.7	47.5	13.4	13.7
Monounsaturated fatty acid	14.5	26.0	23.9	25.3
Polyunsaturated fatty acid (PUFA)	37.3	21.7	52.8	54.2
n6 -PUF / n3 -PUFA	1.5	1.4	19.1	14.1
PUFA / SFA	0.9	0.5	3.9	4.0
UFA / SFA	0.2	1.0	5.7	5.8
Total FA	93.5	95.2	90.0	93.2

${}^{*}$ C: control concentrate. REO: concentrate enriched with rosemary essential oils.

### 2.2 Milk control and analysis

Individual milk yield was assessed at weekly intervals over the first 8 weeks of lactation. During each control, prior to distributing feeds, the lambs were separated from their mothers for 12 h. Subsequently, the ewes were manually milked in the morning at 06:00, and the collected milk volume was recorded and multiplied by 2 to calculate the total daily milk production (van der Linden et al., 2010). For chemical analysis, 50 mL individual milk samples were obtained from all 48 ewes at each control, with the addition of potassium dichromate as a preservative for storage. The fat and protein contents of the milk were determined using an integrated milk analyzer (Combifoss 5300, Foss Electric, Hillerød, Denmark). Additionally, for the last evaluation, individual milk samples (one per animal) were preserved at - 20 °C until the analysis of the fatty acid (FA) composition for all 48 ewes.

### 2.3 Fatty acid analysis

For the analysis of FA, the feed samples were subjected to Soxhlet extraction, while the milk samples underwent lipid separation following the method outlined by Luna et al. (2005). Both groups of FAs were converted into methyl esters through base-catalyzed methanolysis of the glycerides using KOH, following the UNE-EN ISO 5509:2000 standard method. The fatty acid methyl esters were then detected using a gas chromatograph with a flame ionization detector (GC-FID, Bruker 436 gas chromatograph, Bruker, the Netherlands). This chromatograph was equipped with a capillary column composed of bis cyanopropyl polysiloxane (BR2560 WCOT 100 m, 0.25 mm inner diameter (ID), and 0.20 µ m film thickness; Bruker Chemical Analysis BV, the Netherlands). Helium served as the carrier gas, with a flow rate of 1 mL min ${}^{-1}$ . The inlet and detector temperatures were maintained at 250 and 300 °C, respectively. The temperature program used was as follows: the initial temperature was held at 100 °C after injection and then programmed to increase at a rate of 1.5 °C min ${}^{-1}$ to 170 °C (where it was held for 15 min). Subsequently, it increased at a rate of 0.5 °C min ${}^{-1}$ to 180 °C (held for 2 min) and finally increased at a rate of 10 °C min ${}^{-1}$ to 215 °C (held for 20 min). The injection volume was 1.0 µ L. Fatty acids were identified by comparing their retention times with those of a standard FA mixture (Sigma-Aldrich, Madrid, Spain). The individual fatty acid contents were expressed as percentages of the methyl esters of each FA in relation to the total composition (% of the total fatty acid methyl esters – FAME).

### 2.4 Statistical analysis

The experimental design followed a 2 × 2 factorial design involving two forms of forage (oat hay and oat silage) and two types of concentrates (control concentrate and concentrate containing REO). Statistical analyses were conducted using SAS version 8.2. A fixed-effect full-factorial analysis of variance (ANOVA) with a 2 × 2 design was employed to assess variables related to milk composition, milk FA profile, and blood profile. This analysis was performed using the general linear model (GLM) procedure of SAS. For milk production, a repeated-measure ANOVA was conducted using the MIXED procedure of SAS. The model incorporated REO supply, forage type, week, and all potential interactions among them as fixed effects, with ewe status considered a random effect. The significance level was set to 0.05, and trends were considered for P values falling between 0.05 and 0.10.

**Table 2:** Effect of the inclusion of rosemary essential oils (REO) and type of forage (F) in the diet on ewe milk production and composition.

	Treatments ${}^{a}$				SEM	P value ${}^{b}$		
	H-C	H-REO	S-C	S-REO		REO	F	REO ${}^{*}$ F
Daily milk yield (mL d ${}^{-1}$ )	590	547	521	496	35.9	NS	0.07	NS
Milk composition (g L ${}^{-1}$ )								
Fat	66.3	60.9	69.6	60.4	0.50	0.07	NS	NS
Protein	52.0	54.1	59.2	58.1	0.24	NS	0.05	NS
Fat and protein yield (g d ${}^{-1}$ )								
Fat	38.5	32.9	37.1	29.7	3.58	0.07	NS	NS
Protein	30.4	29.5	30.8	28.7	2.13	NS	NS	NS

${}^{a}$ H-C: ewes fed hay and control concentrate. H-REO: ewes fed hay and concentrate enriched with REO. S-C: ewes fed silage and control concentrate. S-REO: ewes fed silage and concentrate enriched with REO. ${}^{b}$ SEM: standard error of the means. F: forage-type effect (silage vs. hay). REO: REO effect. REO ${}^{*}$ F: interaction between REO and the forage type. NS: not significant ( P>0.05 ).

## 3 Results

The interaction between forage type and REO supplementation as well as the forage type × supplement × week interactions were not significant ( P>0.05 ); hence they do not figure in the tables.

### 3.1 Milk production and composition

**Figure 1:**
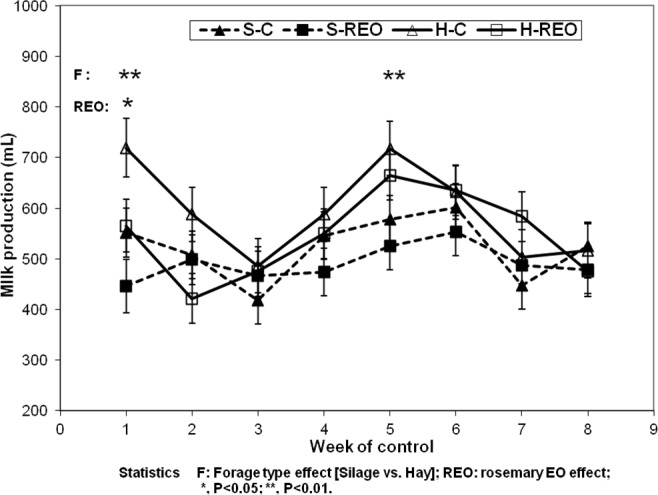
Evolution of the milk production for sheep fed hay and control concentrate (H-C); hay and concentrate enriched with REO (H-REO), silage and control concentrate (S-C); and silage and concentrate enriched with REO (S-REO).

Table 2 reports the milk production, which varied between 0.5 and 0.6 L d ${}^{-1}$ . The milk yield was affected by week ( P<0.001 ), showing a typical lactation curve (Fig. 1). The decrease in milk yield during the third week of lactation could be related to the climatic condition showing a great temperature decrease in this period.

The milk peak varied according to the type of forage. For ewes fed oat hay, the peak was in the fifth week, while for the silage forage, the peak was in the sixth week (Fig. 1). The rosemary EO supply decreased the milk yield, although not significantly ( P>0.05 ), whereas the forage form showed a tendency to increase the milk yield in hay rather than on silage groups. As depicted in Fig. 1, significant differences between the forage types were only observed in weeks 1 and 5 (642 vs. 499 mL and 692 vs. 552 mL for the hay and silage groups, respectively; P<0.01 ). Likewise, a significant difference in the milk yield between the concentrate types was only observed in the first week (506 vs. 636 mL for the REO and control groups, respectively; P<0.05 ).

The REO concentrate did not affect ( P>0.05 ) the fat and protein content (g L ${}^{-1}$ ) and the protein yield (g d ${}^{-1}$ ). However, the REO dietary incorporation tended to decrease ( P=0.07 ) the milk fat yield (38 vs. 32 g d ${}^{-1}$ for the control and REO groups, respectively), which is related to the slightly lower milk yield. On the other hand, the forage form did not affect ( P>0.05 ) the fat content or the fat and protein yield. However, the milk protein content was higher for the silage groups (58.7 vs. 53.1 g L ${}^{-1}$ for the silage and hay groups, respectively; P<0.05 ).

### 3.2 Milk fatty acid profile

The milk FA profile of ewes is presented in Table 3. No interaction between the forage form and REO supply was found ( P>0.05 ). Except for the C18:3 n-3 , the intake of REO did not affect ewe milk's individual FA values (Table 3). However, the milk fat of REO ewes contains lower percentages of PUFA and n-6 PUFA but a higher percentage of n-3 PUFA, especially C18:3 n-3 ; consequently, this resulted in a lower n6/n3 ratio ( P<0.001 ; Table 4).

The forage form has significantly affected the milk FA profile (Tables 3 and 4). In fact, the milk FA of ewes receiving hay was richer ( P<0.05 ) in pentadecanoic acid (C15:0) and n-6 PUFA, while the milk FA of ewes receiving the silage was richer ( P<0.05 ) in the C18:3 n-3 (0.55 vs. 0.45 for the silage and hay groups, respectively), long-chain FA, and n-3 PUFA. The n-6/n-3 was lower ( P<0.01 ) for the silage group than the hay group.

**Table 3:** Fatty acid profile (% of the total FAME) of ewe's milk.

	Treatments ${}^{a}$				SEM	P value ${}^{b}$		
	H-C	H-REO	S-C	S-REO		REO	F	REO ${}^{*}$ F
Butyric acid (C4:0)	1.28	1.61	1.55	1.56	0.184	NS	NS	NS
Caproic acid (C6:0)	1.85	1.96	1.69	1.70	0.208	NS	NS	NS
Caprylic acid (C8:0)	2.34	2.32	1.86	1.82	0.272	NS	NS	NS
Capric acid (C10:0)	7.55	7.37	5.83	5.95	0.873	NS	NS	NS
Lauric acid (C12:0)	4.49	4.37	3.52	3.68	0.463	NS	NS	NS
Myristic acid (C14:0)	11.0	11.4	10.4	10.9	0.66	NS	NS	NS
Pentadecanoic acid (C15:0)	1.46	1.32	1.15	1.26	0.071	NS	${}^{*}$	NS
Palmitic acid (C16:0)	26.1	26.6	28.8	28.2	1.14	NS	NS	NS
Palmitoleic acid (C16:1)	1.46	1.36	1.66	1.61	0.14	NS	NS	NS
Margaric acid (C17:0)	1.00	1.01	0.92	1.18	0.085	NS	NS	NS
Margaroleic acid (C17:1)	0.43	0.38	0.35	0.42	0.028	NS	NS	NS
Stearic acid (C18:0)	10.2	10.0	10.2	10.8	0.74	NS	NS	NS
Oleic acid (C18:1 n-9 )	25.1	25.2	25.6	26.1	1.85	NS	NS	NS
Vaccenic acid (C18:1: n-7 , VA)	0.44	0.38	0.44	0.35	0.035	${}^{*}$	NS	NS
Linoleic acid (C18:2 n-6 )	2.81	2.58	2.79	2.09	0.138	NS	${}^{*}$	NS
α -Linolenic acid (C18:3 n-3 )	0.44	0.46	0.64	0.75	0.051	${}^{*}$	${}^{***}$	NS
γ -Linolenic acid (C18:3 n-6 )	0.27	0.21	0.14	0.27	0.104	NS	NS	NS
Rumenic acid (C18:2c9-t11)	0.49	0.36	0.45	0.41	0.056	NS	NS	NS
γ -Adoleic acid (C20:1)	0.13	0.09	0.14	0.10	0.023	NS	NS	NS
Arachidonic acid (C20:4 n-6 )	0.37	0.27	0.35	0.25	0.041	${}^{**}$	NS	NS
C20:5 n-3 (EPA)	0.04	0.03	0.04	0.04	0.01	NS	NS	NS
Adrenic acid (C22:4 n6 )	0.048	0.034	0.031	0.01	0.045	NS	NS	NS
C22:5 n-3 (DPA)	0.17	0.15	0.17	0.15	0.017	NS	NS	NS
C22:6 n-3 (DHA)	0.04	0.05	0.06	0.06	0.010	NS	NS	NS

${}^{a}$ H-C: ewes fed hay and control concentrate. H-REO: ewes fed hay and concentrate enriched with REO. S-C: ewes fed silage and control concentrate. S-REO: ewes fed silage and concentrate enriched with REO. ${}^{b}$ SEM: standard error of the means. F: forage-type effect (silage vs. hay). REO: REO effect. REO ${}^{*}$ F: interaction between REO and the forage type. NS: not significant ( P>0.05 ). ${}^{*}$ P<0.05 ; ${}^{**}$ P<0.01 ; ${}^{***}$ P<0.001 .

## 4 Discussion

### 4.1 Milk production and composition

The daily milk production and the milk yield evolution and composition were in agreement with the results of Maamouri et al. (2011) for the same breed. The lack of an effect of the addition of REO on milk production and fat content is in line with previous studies where milk yield and composition were unchanged by EO supply (Benchaar et al., 2008; Giannenas et al., 2011; Tager and Krause, 2011). Also, Kholif et al. (2021) showed that milk fat content was not affected by supplementation with EO. In contrast to the present results, Kalaitsidis et al. (2021) recorded a significant improvement in milk yield and fat (6.39 vs. 5.95 %) with a mixture of oregano and thyme essential oils. The same tendency was observed with *Moringa oleifera* essential oil in ewe's milk and fat content (Selmi et al., 2020). In addition, Dorantes-Iturbide et al. (2022) recorded higher milk production, protein, and lactose content in response to EO supply. Similarly, Chiofalo et al. (2010) observed a positive effect of rosemary extract supplementation on milk production and composition: they attributed this improvement to the presence of phenolic compounds. Furthermore, Smeti et al. (2015) showed higher milk production for lactating goats receiving REO or rosemary leaves than control. These different results could be due to the aforementioned authors using rosemary leaves instead of REO.

**Table 4:** Group of FA and nutritional indices of ewe's milk.

	Treatments ${}^{a}$				SEM	P value ${}^{b}$		
	H-C	H-REO	S-C	S-REO		REO	F	REO ${}^{*}$ F
Short-chain FA	13.02	13.25	10.93	11.03	1.44	NS	NS	NS
Medium-chain FA	16.98	17.10	15.09	15.79	1.107	NS	NS	NS
Long-chain FA	37.48	37.71	40.94	40.29	1.201	NS	${}^{*}$	NS
SFA	67.49	68.06	66.95	67.11	1.920	NS	NS	NS
Monounsaturated FA	27.76	27.75	28.39	28.79	1.782	NS	NS	NS
PUFA	4.75	4.19	4.67	4.10	0.216	${}^{**}$	NS	NS
n-6 PUFA	3.57	3.14	3.32	2.65	0.167	${}^{**}$	${}^{*}$	NS
n-3 PUFA	0.69	0.70	0.91	1.05	0.067	NS	${}^{***}$	NS
n-6/n-3	5.20	4.62	3.96	2.56	0.329	${}^{*}$	${}^{***}$	NS
PUFA / SFA	0.069	0.065	0.066	0.065	0.005	NS	NS	NS
UFA / SFA	0.50	0.49	0.49	0.49	0.04	NS	NS	NS

${}^{a}$ H-C: ewes fed hay and control concentrate. H-REO: ewes fed hay and concentrate enriched with REO. S-C: ewes fed silage and control concentrate. S-REO: ewes fed silage and concentrate enriched with REO. ${}^{b}$ SEM: standard error of the means. F: forage-type effect (silage vs. hay). REO: REO effect. REO ${}^{*}$ F: interaction between REO and the forage type. NS: not significant ( P>0.05 ). ${}^{*}$ P<0.05 ; ${}^{**}$ P<0.01 ; ${}^{***}$ P<0.001 .

Regarding the type of forage, milk yield and composition showed significant differences when produced in different green forages (Bonanno et al., 2016; Cabiddu et al., 2022). Previous studies on Sicilo-Sarde dairy sheep comparing oat silage or oat hay to prairie grazing observed that green pasture forage (prairie) resulted in a higher milk yield than silage and hay (Maamouri et al., 2019; Nasri et al., 2019). Furthermore, the recorded milk yield was higher for the cited work than that recorded in the current study; in addition to the low genetic potential of the herd due to inbreeding, the type of concentrate and possible differences in the maturing stage of forage and quality of forage can be the causes of such differences.

### 4.2 Milk fatty acid profile

The fatty milk higher concentrations of palmitic acid (C16:0), oleic acid (C18:1), stearic acid (C18:0) and myristic acid (C14:0) are in agreement with the general tendency reported for dairy sheep (Bonanno et al., 2016; Maamouri et al., 2019; Yonjalli et al., 2020). Except for the C18:3 n-3 , which was improved by the REO supply, there was a difference in the individual FA proportions related to the REO intake; this result is in agreement with other studies on sheep dairy products (milk, cheese and yoghurt) using a mixture of oregano and thyme essential oils (Kalaitsidis et al., 2021) or in sheep meat with cinnamaldehyde, garlic or juniper berry EO (Chaves et al., 2008). Both studies reported that the overall fatty acid profile was not modified by any EO supplementation. Also, and in concordance with the present results, Benchaar et al. (2008) observed a similar impact of terpenes and EO on the milk and meat fatty acid profiles of dairy cows. However, Selmi et al. (2020) recorded a significant effect of *Moringa oleifera* EO on the FA profile of Sicilo-Sarde ewe milk, whereas Yonjalli et al. (2020) showed no change for palmitic acid with tannin extract given that the content of this FA in milk is related to its de novo synthesis, and the stearic acid in milk was significantly affected by tannin extract. Curiously, the supplementation by the same REO largely affected the lamb meat FA profile (Smeti et al., 2018), which could be due to the different used doses of the EO in both studies. The lack of an effect on the FA profile of the EO supply can be related to the ability of ruminal microorganisms to adapt to EO intake and/or to modify the chemical structure of some terpenoids (Malecky and Broudiscou, 2009). This reason confirmed the effect of the polyphenol content of tannin on the milk fatty acid profiles of goats (do Nascimento et al., 2022).

The forage form has significantly affected the milk FA profile. These results are in agreement with previous studies carried out on the same breed and similar forages (Atti et al., 2006). In this sense, Dervishi et al. (2012) found that the forage type affected the long-chain saturated fatty acid content, with higher percentages during grazing than in hay feeding treatments. The variation of the FA profile in relation to a farming system, forage intake in particular, was shown (Agradi et al., 2020; Cabiddu et al., 2021). As we observed, a greater C18:3 n-3 content in milk of silage groups compared to hay ones was previously recorded (Atti et al., 2006; Dervishi et al., 2012). The silage is greener than hay and is nearer to green forage rich in C18:3 n-3 than hay, resulting in a higher PUFA n-3 and then a lower PUFA n-6/n-3 ratio, itself lower with REO intake. Hence, this combination of silage and REO could be beneficial for human health. The long-chain PUFA n-3 plays an important role in the prevention and treatment of certain diseases, such as hypertension, cardiovascular diseases, diabetes, and cancers. The intake of foods with a low PUFA n-6/n-3 ratio was recommended by nutritionists to reduce the risk of these diseases in humans (EFSA, 2009). However, in the current study PUFA, the n-6/n-3 ratio of fatty milk was in the recommended norm ( <4 ) for human health in both silage groups. This ratio did not deviate too far from the norms for the hay group either.

## 5 Conclusions

The inclusion of REO in the diet of lactating ewes did not affect the daily milk yield or the milk composition (fat and protein content). However, it increased the n-3 PUFA significantly and decreased the n-6/n-3 ratio for both forms of forage. Rosemary essential oil could be recommended as a supply in dairy ewes fed silage to improve the quality of their milk for human consumption. Further studies should focus on an adequate level of REO inclusion as well as isolating bioactive components of REO in order to improve the ewes' dairy performances.

## Data Availability

The original data of the paper are available upon request to the corresponding author.
